# Emergency Medicine Resident Orientation: How Training Programs Get Their Residents Started

**DOI:** 10.5811/westjem.2016.10.31275

**Published:** 2016-11-15

**Authors:** Jillian McGrath, Michael Barrie, David P. Way

**Affiliations:** The Ohio State University College of Medicine, Department of Emergency Medicine, Columbus, Ohio

## Abstract

**Introduction:**

The first formal orientation program for incoming emergency medicine (EM) residents was started in 1976. The last attempt to describe the nature of orientation programs was by Brillman in 1995. Now almost all residencies offer orientation to incoming residents, but little is known about the curricular content or structure of these programs. The purpose of this project was to describe the current composition and purpose of EM resident orientation programs in the United States.

**Methods:**

In autumn of 2014, we surveyed all U.S. EM residency program directors (n=167). We adapted our survey instrument from one used by Brillman (1995). The survey was designed to assess the orientation program’s purpose, structure, content, and teaching methods.

**Results:**

The survey return rate was 63% (105 of 167). Most respondents (77%) directed three-year residencies, and all but one program offered intern orientation. Orientations lasted an average of nine clinical (Std. Dev.=7.3) and 13 non-clinical days (Std. Dev.=9.3). The prototypical breakdown of program activities was 27% lectures, 23% clinical work, 16% skills training, 10% administrative activities, 9% socialization and 15% other activities. Most orientations included activities to promote socialization among interns (98%) and with other members of the department (91%). Many programs (87%) included special certification courses (ACLS, ATLS, PALS, NRP). Course content included the following: use of electronic medical records (90%), physician wellness (75%), and chief complaint-based lectures (72%). Procedural skill sessions covered ultrasound (94%), airway management (91%), vascular access (90%), wound management (77%), splinting (67%), and trauma skills (62%).

**Conclusion:**

Compared to Brillman (1995), we found that more programs (99%) are offering formal orientation and allocating more time to them. Lectures remain the most common educational activity. We found increases in the use of skills labs and specialty certifications. We also observed increases in time dedicated to clinical work during orientation. Only a few programs reported engaging in baseline or milestone assessments, an activity that could offer significant benefits to the residency program.

## INTRODUCTION

Emergency medicine (EM) residency programs commonly offer dedicated curricula designed for orientation of beginning residents. An orientation curriculum was first developed for incoming EM residents at the University of Cincinnati in 1976.^1^ Major objectives of that first orientation were to identify and delineate the subject matter of EM and to review the basic elements of EM. In 1995, Brillman et al. surveyed EM residency program directors regarding composition of orientation curricula. At that time, 93% of EM programs offered an orientation program. Orientation consisted mainly of lectures and certification courses, had variable lengths, composition, goals, and associated courses, and very few programs offered procedural labs or special skills training sessions (2–11%).^2^ More recently, Lucas et al. described a redesigned resident orientation curriculum using the Kern model of curriculum development.^3^,^4^ Components of their redesigned curriculum included instruction on administrative procedures and policy, skills training, instruction on medical knowledge, setting expectations for learning, introductory performance assessment, and socialization. Min et al. also described an optional introductory clinician development course (intern “boot camp”) prior to the start of residency, which focused on core medical content, common patient presentations, basic procedural skills instruction and supervised clinical shifts.^5^ Both Lucas and Min asked new EM residents to rank components of their curriculum in terms of perceived “helpfulness.”

Since 1995 there have been no general descriptions or studies describing EM orientation practices throughout the U.S. Additionally, we found no standards to guide program development. The literature is rich, however, with conversations about bridging the gap between undergraduate medical education (UME) and graduate medical education (GME),^6^ which include specifics about assessing medical students and medical graduates at these critical stages of professional development (e.g. Entrustable Professional Activities, and American Council on Graduate Medical Education [ACGME] Milestones).^7^–^9^ There is less in the literature about how orientation programs contribute to the transition from UME to GME, particularly in the specialty of EM.

The purpose of this project was to profile the current state of orientation programs for entering EM residents across the U.S. At the outset, we anticipated an increase in the number of formal orientation programs and also predicted that we would find considerable variability in program characteristics, length, and goals. Further, we expected to find that residency programs had increased their use of benchmark assessments for incoming interns to determine where they were in their progress towards achieving the ACGME milestones. Finally, we hoped to find significant innovation in program activities and assessments that might be generalizable to others.

## METHODS

### Study Participants

We surveyed the EM residency program directors of all ACGME-accredited programs in the U.S. Survey participants were identified through three different residency program directories: The Society for Academic Emergency Medicine Residency Directory,^10^ the American College of Emergency Physicians Directory of Approved ACGME Residencies,^11^ and the American Medical Association’s FREIDA Online® Services.^12^

### Instrument development

We adapted our survey instrument from one used by Brillman (1995).^2^ Adaptations included changes to the types of questions asked, and the addition of questions regarding contemporary teaching methods. Instead of open-ended questions to gather program information, we asked respondents to choose items from checklists with instructions to select all that apply. We also added questions about the use of high-fidelity simulation, simulated patient encounters, and social activities. Finally, unlike Brillman’s survey, we asked respondents to give us an idea of the overall program structure by estimating the percentage of time allocated to each of 10 types of program activities. The survey was developed collaboratively among former and current residency program directors and associate program directors, under the direction of a survey development specialist. Two of the developers have designed and administered an orientation program for our local residency. All developers have participated in an orientation program as residents.

Survey developers were presented with a draft survey derived from the Brillman article. They were asked to add, modify, or delete items to create an instrument that contained only items they believed were important for profiling a modern residency orientation program. The subsequent results were fine-tuned into proper survey format and then presented to the developers as a pilot, which led to an additional round of modifications.

The final instrument contained 18 items: 13 checklist items, one multiple choice, one fill in the blank and three open-ended comment items. To shorten the survey administration time, each of the checklist items was preceded with a skip logic question, which is a yes vs. no filter item that directs the respondent only to applicable subsequent checklists.

### Survey Implementation

We used the Dillman tailored design method (TDM) for electronic (e-mail) surveys to guide this national study.^13^ Notices about the study were sent in advance to residency directors. Email communications were personalized. The cover letter and survey were delivered within three days of the initial notice. Respondents were offered an alternative method for sending back their responses. Finally, including the initial notice, we contacted program directors up to five times with reminder notifications or personal requests to complete the survey. Email addresses were verified and updated at all stages of survey implementation. Our institution’s human subjects review board approved this survey project.

### Data Analysis

We analyzed electronic survey data with IBM-SPSS for Windows, Version 22.0.^14^ We compared the respondents and non-respondents on demographic characteristics to check that our respondent data were representative of the population using chi-square tests of proportions (*X*^2^). The program demographics that we tested for bias included region of the country (Northeastern, Central, Southern, and Western); program length (three- or four-year program); and program size (number of residents) by percentile rank (1–25th percentile, 26th-50 percentile, 51–75th percentile, and 76–99th percentile). We used descriptive statistics to profile the orientation programs for EM medicine residency programs. (Note: Since 1995, three-year programs that start in the postgraduate year 2 have been phased out.)

## RESULTS

The overall survey return rate was 63% (105 of 167). The respondent sample was evaluated for representativeness using chi-square tests of proportion (Table 1). Survey participants were representative of the population of residency program directors in EM with regard to program size, program length, and region of the country.

All but one of the EM residency program director respondents said that they conduct intern orientations (99%; or 104 of 105). Orientation programs were most frequently sponsored by the Department of EM (97%), but some programs obtain additional sponsorship through the following: academic health centers (AHCs) (59%), medical schools (14%), or other affiliated hospitals (12%). One program said that their orientation program was sponsored through their Graduate Medical Education Office and that most of the orientation activities were shared with interns from other specialties’ residency programs.

The length of EM orientation programs averaged 22 days (SD=11.8). Residents spent an average of 8.9 days (SD=7.3) of clinical orientation, i.e. clinical work in the ED. Non-clinical activities accounted for 13.2 orientation days (SD 9.3).

**Figure 1:**
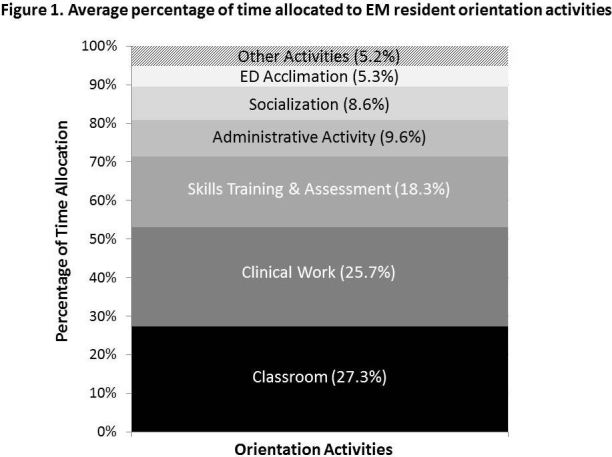
Average percentage of time allocated to emergency medicine resident orientation activities.

We asked directors to estimate how they allocated their orientation time across various activities (Figure 1). Directors reported that about a quarter of their time was allocated to classroom didactics (27%), and a quarter to clinical work, including pediatric clinical work (23%). The other half was mostly comprised of skills training and assessment (18%), administrative activities (10%), socialization (9%), ED acclimation (5%), and miscellaneous other activities (5%).

The most frequently expressed goals of orientation programs were an opportunity for interns to get to know each other (98%), familiarization with hospital and departmental policy (95%), acclimating to a new ED (93%), opportunity to get to know other members of the department (91%), and completion of administrative tasks (89%). Less frequently expressed goals were review of skills or medical knowledge learned in medical school (59% and 55%), baseline assessment of clinical skills and medical knowledge (54% and 50%), and other purposes, which included additional baseline assessment and certification courses (11%) (Table 2).

**Figure 2:**
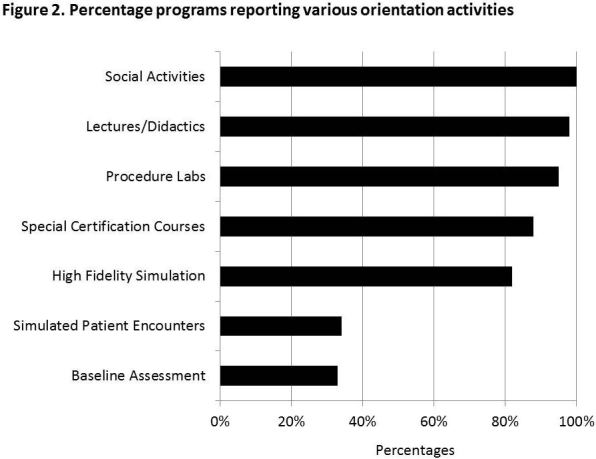
Percentage programs reporting various orientation activities.

Specific orientation activities offered by programs included social activities (100%), lectures/didactic sessions (98%), procedure labs (95%), special certification courses (87%), high-fidelity simulation (82%), simulated patient encounters/objective structured clinical examination (OSCE) (34%), and baseline assessment (33%) (Figure 2).

The most frequent topics included in lectures or didactic sessions were use of the electronic medical record (90%), physician wellness (75%), and chief complaint-based lectures (72%). Table 3 lists the other topics covered by didactic sessions.

Specific procedural or skill sessions offered by programs included ultrasound (94%), airway management (91%), vascular access (90%), suturing/wound management (77%), splinting (67%), trauma-related (62%), cadaver-based lab (25%), Head, eye, ears, nose and throat emergencies (HEENT) (17%), animal-based lab (12%), dental lab (8%), and other skills (10%) (Table 4).

Specialized certification courses were offered by 88% of programs. Specific specialized courses offered during the orientation curriculum were ACLS (77% of all programs), PALS (74%), ATLS (68%), NRP or other neonatal courses (27%), and other specialized courses (12%).

The most frequent social activities offered by programs were social events for both EM residents and faculty (87%), team-building activities (55%), and social events for EM interns only or EM residents only (40% and 40%). Twenty-eight percent of programs offer a formal retreat that occurs off-site and 18% offer social events that include other ED personnel such as nursing or staff.

For programs that perform baseline assessment of new EM residents, baseline assessment practices focused on medical knowledge (79%), patient communication (49%), history-taking skills (42%), physical exam skills (36%), EKG interpretation (36%), emotional intelligence/personality assessments (27%), radiology interpretation (18%), learning style tests, evidence-based medicine (EBM) knowledge (9%), or other skills such as Level 1 Milestones or procedural skills (12%).

We identified several themes through analysis of verbatim comments. First, EM residency program directors appreciated having time within an orientation curriculum for bonding and socialization. They also valued dedicated time to introduce their care delivery system and expectations for the program. However, there was an expressed desire to further streamline administrative requirements and tasks, continue to move away from lecture-based curriculum while placing more emphasis on interactive didactics (small groups, procedural or skills labs, simulation, and OSCEs), and incorporate more assessment of baseline skills.

Survey respondents described innovations such as active learning experiences that include procedural assessment, and simulation experiences that involve breakdowns of critical steps and opportunities to train and remediate specific steps. Programs also included many special topics such as EBM skills, patient safety and quality, consultation skills, crew resource training, electronic communication-social media use, and work-life balance. Among programs that conducted baseline milestone assessment, some confirm that all Level 1 Milestones are met, while others evaluate only select Level 1 Milestones.

## DISCUSSION

In comparison to the 1995 survey by Brillman et al, slightly more EM programs are offering a formal orientation curriculum (2014= 99% vs. 1995=93%). Orientations now average 22 days, a 57% increase over the 14 days reported by Brillman in 1995. The difference appears to come from the 8.9 days, on average of additional clinical time working in the ED, almost triple that of Brillman’s reported 2.4 days. Similar to the 1995 survey, the activity with the most dedicated time during orientation was lecture-based didactics (2014= 34 h vs. 1995= 35 h). However, we note an overwhelming increase in the number of programs that offer procedure labs and specialty sessions during their nonclinical orientation (2014= 95% vs. 1995= 52%). As Brillman reported in 1995, EM programs continue to offer specialized courses during orientation (ACLS: 2014= 74% vs. 1995= 84%; and ATLS: 2014= 65% vs. 1995= 68%). Considerably more programs are now offering a PALS course (2014= 71% vs. 1995= 39%). We report an increase in the proportion of dedicated time applied to formal clinical orientation (ED clinical work), 44% in 2014 vs. 17% in 1995, as well.

The three-fold increase in ED clinical work during orientation between 1995 and 2014 is perhaps best explained by the survey participants’ responses to the “purpose” for orientation. Most of the purposes provided seem to involve enculturation: Getting to know one another, familiarizing interns with the hospital and department policies, acclimation to a new ED, getting to know members of the department, and team building. Since only 33% of respondents said that they include formal baseline assessment during orientation, an alternative explanation is that the additional ED clinical work is designed for informal assessment of an intern’s baseline clinical skills.

Innovations described by respondents include an increasing number of specialty topics and sessions, expanded active learning experiences, and incorporation of introductory assessment and baseline EM milestone assessment. With the increased focus on competency-based assessment introduced by the ABEM/ACGME Milestone Project,^9^ we were surprised to observe that only 32.7% of program directors reported the incorporation of baseline assessment of clinical skills during orientation. We speculate that this survey project, conducted in late 2014, was out ahead of residency programs’ implementation of formal milestone assessments (such as the one described by Hauff, et al.),^15^ and that the landscape has likely shifted from informal to formal assessment over the past two years.

Documentation of medical student progress towards Level 1 Milestones could offer significant benefits to residents, their residency programs, and ultimately their patients. Deficiencies could be identified and remediated earlier, or customized learning plans based on milestone achievement could be developed. Competency-based assessment that document milestone progress or measure attainment of “Entrustable Professional Activities” are being developed and are beginning to surface in the literature.^7^,^15^,^16^ However, when assessments should be conducted and who should be responsible for assessment, whether it should be UME or GME programs, are questions that remain unanswered.^7^ Future research should contribute to identifying “best practices” for improving the learner “hand-off” process from UME to GME.

## LIMITATIONS

We demonstrated that our respondents were representative of the population as a whole, but because we did not receive a survey from every program, generalizability to all programs is not assured. Additionally, we should note the limitations common to survey research. First is the potential that selection bias occurred, which in our case would have been the tendency for residencies with no orientation program to have avoided participation in the survey. Second is the potential for recall bias among those who completed the survey. Finally, we cannot be certain that we captured the rich detail of every residency orientation program. By seeking a general profile of residency orientation, some unique and creative program details may have remained undetected.

## CONCLUSION

Since the last national survey of EM residency program directors about their orientation programs, much has changed. Now, nearly every program has an established orientation program for incoming residents. Overall, the duration of orientation has increased by almost 60%, which is primarily attributable to increases in dedicated clinical work during orientation. The most common activities remain didactic sessions and social activities, but with improvements in technology and simulation, there has been an increase in skill training sessions. A minority of programs implement baseline assessments of their learners, which is an opportunity for programs to develop early interventions for incoming residents not meeting minimum expectations.

## Figures and Tables

**Table 1:** Demographic profile of emergency medicine residency programs in the U.S. by survey respondents and non-respondents: program size, program length, and region of the country.*

Demographics	Respondents	Non-respondents	Total
Program size			
Below 25th percentile	14 (52%)	13 (48%)	27 (17%)
25th – 50th percentile	37 (65)	20 (35)	57 (36)
51th – 75th percentile	24 (62)	15 (39)	39 (24)
Above 75th percentile	27 (73)	10 (27)	22 (23)
Data unavailable			7 (4)
	*X*^2^= 3.13, df=3, p= .37		
Program length			
3-Year	83 (62%)	50 (38%)	133 (80%)
4-Year	21 (67)	10 (32)	31 (47)
Data unavailable			3 (2)
	*X*^2^= 0.31, df=1, p= .68		
Region			
Northeast	33 (60%)	22 (40%)	55 (33%)
Central	26 (65)	14 (35)	40 (24)
South	30 (63)	18 (38)	48 (29)
West	16 (67)	8 (33)	24 (14)
	*X*^2^= 0.42, df=3, p= .94		
Total	105 (63%)	62 (37%)	167 (100%)

* The authors surveyed residency program directors of 167 emergency medicine residency programs in the United States. The respondents of the survey are profiled demographically using residency program characteristics: program size (number of residents), program length (three- or four-year program), and region of the country. Chi-square tests of proportion (*X*^2^) are used to evaluate whether the sample obtained is representative of the population at large.

**Table 2:** Frequency and percentages of purposes served by EM orientation programs as reported by 104 U.S. residency program directors (Directors were permitted to select more than one purpose.)

	Frequency	Percent
Getting to know each other	103	99.0
Familiarizing interns with hospital and department policies	99	95.2
Acclimation to a new emergency department	97	93.3
Getting to know members of the department	95	91.3
Administrative tasks and chores	93	89.4
Promoting positive environment	90	86.5
Team building	85	81.7
Teaching new skills	78	75.0
Teaching new knowledge	76	73.1
Earning additional credentials such as ACLS, ATLS, etc.	70	67.3
Reviewing skills learned in medical school	61	58.7
Baseline assessment of clinical skills	57	54.8
Reviewing medical knowledge learned in medical school	57	54.8
Baseline assessment of medical knowledge	52	50.0
Other purpose not listed	12	11.5

*ACLS,* advanced cardiac life support; *ATLS,* advanced trauma life support

**Table 3:** Frequency and percentages of topics covered through didactics or lectures during EM orientation programs as reported by 102 U.S. residency program directors (Directors were permitted to select more than one topic.)

	Frequency	Percent
Electronic medical record	92	90.2
Wellness	76	74.5
Clinical chief complaint-based lectures	73	71.6
Patient safety/quality	69	67.6
EKG interpretation	67	65.7
Trauma	67	65.7
Nursing integration	60	58.8
Work-life balance	58	56.9
Clinical topic-based lectures	56	54.9
Consultation	55	53.9
Radiology interpretation	51	50.0
Social media	51	50.0
Research	47	46.1
Electronic communication	43	42.2
Coding/billing	46	45.1
Impaired physician	46	45.1
Regulatory/legal	46	45.1
Ethics	38	37.3
EMS	32	31.4
EBM	31	30.4
Culture/diversity	27	26.5
Crew resource management	17	16.7
Other topics not listed	17	16.2
Personal financial	15	14.7
Palliative care/advanced directives	8	7.8

*EKG,* electrocardiogram; *EMS,* emergency medical services; *EBM,* evidence based medicine

**Table 4:** Frequency and percentages of topics covered through procedural skill sessions during EM orientation programs as reported by 99 U.S. residency program directors (Directors were permitted to select more than one topic.)

	Frequency	Percent
Ultrasound	93	93.9
Airway management	90	90.9
Vascular access	89	89.9
Wound management/suturing	76	76.8
Splinting	66	66.7
Trauma-related procedures (e.g. chest tube placement)	61	61.6
Cadaver-based lab	25	25.3
HEENT	17	17.2
Animal-based lab	12	12.1
Dental emergencies	8	8.1
Other skill set not listed	10	10.1
Arthrocentesis		
Pericardiocentesis		
Venous pacing		
Transcutaneous pacing		
OB delivery		
CV insertion		
Decontamination		
Line placement (3)		
Common bedside procedures such as Foley catheters, NG tube placement		
Sexual assault forensic examination		
Slit lamp usage (3)		
Incision and drainage of abscesses		

*OB,* obstetrics; *CV*, central venous; *HEENT,* head ears eyes neck throat
